# Cases of Scrofula Cured by the Xanthoxylum Fraxineum*For this communication we are indebted to an unprofessional gentleman in Mississippi of great respectability and intelligence. Full reliance may be placed upon the accuracy of his statements.

**Published:** 1841-11

**Authors:** 


### Art. III.—*Cases of Scrofula cured by the Xanthoxylum Fraxineum.**

Physicians, I believe, agree, that many of their most valuable remedies were discovered and first used by empirics; and hence the necessary conclusion that the use of a remedy by an empiric is only *prima facie* evidence against its efficiency. It is my wish to direct the attention of physicians to a remedy, which appears to have been used by quacks, with at least apparent success, in one of those diseases which appear to be mingled with the springs of life so perfectly, that palliation is attempted rather than relief. I refer to a shrub, the botanical name of which I do not know. I have heard it called Prickly Ash, Prickly Sumach,† and Tear-blanket. It is very common here in the West, and I believe generally known by one of these names. The case in which this remedy was first used within my knowledge, was that of a negro-woman of mine, some fifty years of age. She had been under the care of a physician for some months, without having derived the

*For this communication we are indebted to an unprofessional gentleman in Mississippi of great respectability and intelligence. Full reliance may be placed upon the accuracy of his statements.

†These are not the same plant. The Xanthoxylum Fraxineum is no doubt the one referred to. The Aralia Spinosa, (Prickly Sumach) we incline to think, possesses no medicinal properties. Y.

least benefit from the usual remedies. The ulcers about the throat had become very deep and large. She was very much emaciated. In short, the physician despaired of her wholly, and suggested the propriety of trying the Prickly Ash, saying that he had heard, when a boy, that it had been used successfully in that disease. He tried it, and in a short time all the urgent symptoms were relieved—the ulcers assumed a healthy appearance and healed up perfectly—the only application to them being a little tallow upon a rag. The disease appeared to have been perfectly eradicated in that case, there having been no symptom of its return during three years.

The next case that I saw was a man some 40 years of age—very spare habit. His family was scrofulous. His throat was much scarred when I saw him in 1840. He said, that for some years previous to 1828, he had been much afflicted with scrofula, not only with the ulceration about the jaws, but with violent pain in the chest, inflammation about the eyes; the water from his eyes was so acrid that it excoriated his cheeks. After having, as he expressed it, "spent all his earnings upon the Doctors without being benefitted at all," he, in 1828, applied to a negro-doctor, who gave him the Prickly Ash; he was entirely cured and remained free from scrofula until his death, last spring.

The next case that I saw, was that of a young lady who had had the advice of the most eminent of our Western physicians; indeed, she had been under the care of one of the most eminent for some years, with scarce even a temporary relief. She had entirely despaired of recovery. I recommended the Prickly Ash; in a few weeks all the swelling about the fauces subsided, the gums and cheeks assumed a healthy hue; she was relieved from the violent headachs to which she had been subject for years. She left the country before her cure was completed, and I understand that some of the symptoms have since returned.

I have been thus particular in describing the cases, in order that those who have better opportunities of forming a corrcct opinion, may decide as to whether there be any hope that this may prove a specific in this horrible disease.

I take a handful of the bark of the stem or root, and boil it in a *new* iron vessel. I let it stand until the iron has blackened the tea. I give it thrice daily—let the patient take as much as the stomach will bear without nausea. A little rhubarb may occasionally be necessary.

I hope that my experience may induce some of the profession to try the remedy. I would suggest the propriety of making an inspissated preparation of the tea after the iron has combined with it, for patients complain very much of the bitter nauseous taste of the tea. P. H. S.

*September*, 1841.

